# Progress toward the tomato fruit cell wall proteome

**DOI:** 10.3389/fpls.2013.00159

**Published:** 2013-05-29

**Authors:** Eliel Ruiz-May, Jocelyn K. C. Rose

**Affiliations:** Department of Plant Biology, Cornell UniversityIthaca, NY, USA

**Keywords:** tomato, fruit, cell wall, proteomics, secretion

## Abstract

The plant cell wall (CW) compartment, or apoplast, is host to a highly dynamic proteome, comprising large numbers of both enzymatic and structural proteins. This reflects its importance as the interface between adjacent cells and the external environment, the presence of numerous extracellular metabolic and signaling pathways, and the complex nature of wall structural assembly and remodeling during cell growth and differentiation. Tomato fruit ontogeny, with its distinct phases of rapid growth and ripening, provides a valuable experimental model system for CW proteomic studies, in that it involves substantial wall assembly, remodeling, and coordinated disassembly. Moreover, diverse populations of secreted proteins must be deployed to resist microbial infection and protect against abiotic stresses. Tomato fruits also provide substantial amounts of biological material, which is a significant advantage for many types of biochemical analyses, and facilitates the detection of lower abundance proteins. In this review, we describe a variety of orthogonal techniques that have been applied to identify CW localized proteins from tomato fruit, including approaches that: target the proteome of the CW and the overlying cuticle; functional “secretome” screens; lectin affinity chromatography; and computational analyses to predict proteins that enter the secretory pathway. Each has its merits and limitations, but collectively they are providing important insights into CW proteome composition and dynamics, as well as some potentially controversial issues, such as the prevalence of non-canonical protein secretion.

## INTRODUCTION

Tomato (*Solanum lycopersicum*), one of the world’s most important horticultural crops, is recognized as the pre-eminent experimental model to study fleshy fruit development, physiology, and pathology (Klee and Giovannoni, 2011; Tomato Genome Consortium, 2012; Seymour et al., 2013). Particular attention has been paid to attributes of tomato fruit that are associated with consumer desirability, such as color, flavor, aroma, and texture, and considerable progress has been made in understanding the molecular processes that underlie these traits. In some cases these are directly and clearly associated with specific biochemical or regulatory pathways that are now well understood, such as the synthesis of pigments, or the gaseous hormone ethylene. However, in other instances they relate broadly to subcellular features whose role in fruit development is highly complex and less well defined. An example of the latter is the intimate, but still poorly understood relationship between fruit texture and the metabolism of the cell wall (CW). This is reflected in an extensive literature, spanning many decades, describing the putative roles of numerous, functionally diverse families of CW resident (i.e., apoplastic, or secreted) proteins and their cognate genes in ripening-related textural changes (Brummell, 2006; Prasanna et al., 2007; Vicente et al., 2007; Payasi et al., 2009; Palma et al., 2011). Progress to date in experimentally addressing the enzymatic basis of CW modification and the relationship with softening has typically been piecemeal, targeting specific activities or individual genes and related proteins. Perhaps the best studied example of such is the pectin degrading enzyme polygalacturonase (PG), whose expression was suppressed in the first commercially available genetically engineered whole food, the Flavr Savr tomato (Smith et al., 1988; Kramer and Redenbaugh, 1994). Since then, other genes encoding CW modifying proteins have been targeted in transgenic tomato fruits in an effort to prevent over-softening and textural deterioration, although mostly without success (Brummell, 2006; Vicente et al., 2007). Such experiments have provided many insights into CW metabolism, but an important outcome has also been the realization that CW disassembly is the consequence of the synergistic actions of many proteins, and that a significant understanding of the dynamics of wall architecture and the mechanistic basis of softening will require a more complete compendium of the CW proteome. This has spurred efforts to study the CW proteome, or “secretome,” of tomato fruit during ripening, as well as during cuticle and CW biosynthesis (Faurobert et al., 2007; Yeats et al., 2010, 2012a,b; Catalá et al., 2011; Palma et al., 2011).

As with any species, genomic and transcriptomic studies of tomato provide an invaluable, if not essential platform for equivalent proteomics analysis and the recent publication of the tomato genome sequence includes more than 700 gene models annotated with CW-related functions (Tomato Genome Consortium, 2012). Transcript analysis of tomato fruit development and ripening showed the differential expression of more than 50 genes associated with CW modification (Tomato Genome Consortium, 2012), while a more detailed analysis of the cell/tissue type-specific transcriptomes of tomato fruit at the maximal stage of growth revealed 253 glycosyltransferases (GTs) and 293 glycosyl hydrolases (GHs) related to CW biogenesis and disassembly, respectively (Matas et al., 2011). Such information, together with a wealth of analytical tools and bioinformatic resources, has laid the foundation for cataloging the tomato fruit CW proteome in a more comprehensive fashion. This will include as assessment of alternate splicing of transcripts to reveal the multiple proteins variants that can result from single genes, as well as the broad range of post-translational modifications (PTMs).

## THE CHALLENGES OF IDENTIFYING FRUIT CW PROTEINS

The pioneering study of the plant CW proteome involved an analysis of cell suspension cultures derived from several plant species, including tomato, that were washed sequentially with buffers of different ionic strengths in order to isolate proteins that were designated as soluble, weakly or strongly bound to the CW (Robertson et al., 1997). Since then, different approaches have been used to characterize the CW proteomes of different tissues from a broad range of plants (Blee et al., 2001; Chivasa et al., 2002; Borderies et al., 2003; Watson et al., 2004; Bayer et al., 2006; Jamet et al., 2006, 2008; Zhu et al., 2006, 2007; Negri et al., 2008; Albenne et al., 2009; Chen et al., 2009; Cho et al., 2009; Millar et al., 2009; Zhang et al., 2010). However, as previously described (Lee et al., 2004; Rose and Lee, 2010), characterization of the plant CW proteome is technically challenging compared to that of other subcellular fractions. Firstly, the “cell wall,” in the context discussed here, is not bound by a distinct membrane that can facilitate isolation, but rather corresponds to the apoplastic continuum and associated extracellular matrix that extends throughout the plant. Attempts to isolate a comprehensive set of wall localized proteins must therefore contend with the conflicting needs to isolate proteins that can be extremely tightly linked to the wall matrix, and thus require harsh treatments to liberate them, as well as proteins that are mobile in the apoplast and are readily lost upon tissue disruption. Any cellular breakage will also immediately result in severe contamination of the protein fraction with cytoplasmic proteins and possibly also those from intracellular organelles (Lee et al., 2004; Feiz et al., 2006). Isolation strategies can therefore be designated as “disruptive,” where researchers must address the challenges of identifying proteins resulting from the inevitable contamination; or “non-disruptive,” where techniques such as vacuum infiltration typically yield a very small fraction of the total CW proteome, and only those protein species that are weakly affiliated with the CW. We also note that even non-disruptive approaches will almost certainly cause some cellular lysis.

In one sense, fleshy fruits provide a “worst case scenario,” as not only are their walls often particularly rich in highly charged anionic pectin polymers that form gels and confound protein isolation, but also the degree of charge and physicochemical properties of the wall matrix change during ripening. This means that the ease of extraction of a particular protein may change substantially at different developmental stages. Thus, a protein may be present and detected in a soluble fraction derived from one developmental stage, but “vanish” from a similar extract from a subsequent developmental stage as it is more tightly bound to the CW and thus less easily extracted. The reverse may also be true and we have observed that this can be a chronic problem for some proteins. In addition, most CW proteins are glycosylated, sometimes to a large degree, which also affects ease of isolation and downstream analysis. This makes accurate quantitative analysis extremely difficult, if not impossible in some cases. We contend that CW proteome studies should be considered as analyses of the “extractome,” rather than the true proteome.

It is important to bear in mind that there is no one perfect approach and that an extensive catalog of the CW proteome requires multiple orthogonal strategies, including techniques that enrich for wall proteins and bioinformatic analyses (Lee et al., 2004; Feiz et al., 2006; Rose and Lee, 2010; Ruiz-May et al., 2012b). Assessment of protein localization *in silico*, based on the predicted presence or absence of subcellular targeting sequences, can provide a valuable tool for biologists. Indeed, current algorithms are generally highly effective; however, they are not perfect predictors (Rose and Lee, 2010) and for a high confidence determination of true wall localization, confirmatory analysis, such as fluorescence protein fusion localization or immunolocalization is essential. It is notable that, to our knowledge, no CW proteome profiling study to date has followed up the identification of a potentially “non-classically secreted CW protein” with such a confirmatory analysis. Until this is done such reports should be treated with great caution.

## INSIGHTS INTO THE TOMATO FRUIT PROTEOME

To date, there have been few systematic studies of the tomato fruit CW proteome compared to those that have targeted *Arabidopsis thaliana* (Nikolovski et al., 2012; Parsons et al., 2012; Zielinska et al., 2012). However, several reports have focused on specific aspects of tomato fruit biology, as summarized below.

Perhaps the most important question in arena of tomato fruit CW proteomics is the relationship between CW resident proteins and the complex textural changes that occur during ripening, which are loosely referred to as “softening” (Vicente et al., 2007; Seymour et al., 2013). One obvious approach is to identify the suites of wall localized proteins that are expressed during ripening, while another is take advantage of the diversity of texture associated phenotypes that are collectively exhibited by different cultivars, and to correlate those differences with patterns of CW protein expression. To this end, Konozy et al. (2013) used a proteomic approach to qualitatively compare the CW proteomes of fruits from three tomato cultivars with distinctly different fruit textural traits. Both non-disruptive and disruptive approaches were used to isolate soluble apoplastic proteins and those that were weakly bound to the CW, respectively. The former used vacuum infiltration-centrifugation of tomato pericarp samples, while the disruptive assay involved pericarp tissue homogenization and consecutive washing of the CW enriched pellet in order to reduce contamination with cytosolic proteins, followed by elution of a CW protein fraction with a buffer containing a moderate salt concentration. A total of 75 proteins were identified, many with a predicted or known CW localization, although no major differences were observed between cultivars. Further experiments will be needed to determine whether any of these CW proteins is responsible for the textural characteristics associated with each cultivar. However, this study represents one of the first efforts to profile the CW proteome of tomato fruit pericarp using sequential extraction approaches that have previously been applied to other plant organs and complex tissues (Watson et al., 2004; Zhu et al., 2006).

In addition to those involved in CW metabolism, substantial numbers of apoplastic proteins and peptides function in plant defense against microbial pathogens, including many of the classical pathogenesis-related (PR) proteins (van Loon et al., 2006; Ferreira et al., 2007; Lagaert et al., 2009; Benko-Iseppon et al., 2010; De-la-Peña and Vivanco, 2010). The susceptibility of ripening fruit to infection can also be influenced by endogenous CW disassembly (Cantu et al., 2008) and so characterization of extracellular proteins in the microenvironment of the infection site may provide insights into the complex factors that affect the nature and timing of the interaction between fruits and pathogens. Shah et al. (2012) used a non-disruptive shotgun proteomics approach to isolate and identify extracellular proteins associated with the infection of tomato fruit by the necrotrophic fungus *Botrytis cinerea*. A total of 558 tomato proteins were identified from the mature green (MG) and red ripe (RR) stage of wild type fruits and those from the non-ripening *ripening inhibitor* (*rin*) mutant, all of which had been inoculated with the fungus. These included proteins belonging to many of the classical PR families, as well as members of diverse protein families such as proteases, protease inhibitors, and peroxidases, revealing a complicated CW proteome cocktail. Interestingly, substantially fewer defense-related proteins were identified in the MG fruit compared with the non-ripening *rin* or RR wild type fruits, although the authors point out that this may reflect differences in tissue homogenization, and thus presumably extractability, in addition to possible difference in the host response to the pathogen. Regardless, this study provided a useful simultaneous qualitative snapshot of the fruit and pathogen CW-related proteomes.

Another important factor that provides a critical barrier against microbial pathogens, as well as protection against pests and abiotic stresses such as desiccation and UV radiation, is the plant cuticle, a specialized lipid rich plant CW that covers the aerial epidermis of land plants (Isaacson et al., 2009; Reina-Pinto and Yephremov, 2009; Yeats et al., 2012b). Tomato fruit are an excellent system in which to identify the proteins involved in cuticle formation and restructuring since, like many fruits, their cuticle is typically much thicker than that of vegetative organs. Yeats et al. (2010) took advantage of this in a study aimed at identifying proteins associated with tomato fruit cuticle biosynthesis. The authors employed a non-disruptive protocol by briefly dipping fruits in an organic solvent, followed by several protein fractionation strategies and two mass spectrometry techniques [LC-ESI-MS/MS (liquid chromatography–electrospray ionization tandem mass spectrometry) and MALDI-TOF/TOF (matrix-assisted laser desorption ionization–time-of-flight tandem mass spectrometer)]. A total of 202 proteins were identified, of which approximately 40% had a predicted N-terminal signal peptide (SP) suggesting targeting to the CW, although the tomato genome sequence was not available at the time of the analysis and so missing N-terminal sequences would likely have resulted in an underestimation. A number of lipid metabolism-related proteins were identified, one of which was a GDSL (Gly-Asp-Ser-Leu)-motif lipase/hydrolase that was recently shown to be cutin synthase, the enzyme that catalyzes the polymerization of cutin monomers at the polysaccharide CW–cuticle interface (Yeats et al., 2012b). This exemplifies the value of CW proteomics for targeting specific biological questions.

Beyond identifying CW protein sequences, a crucial level of information that is slowly starting to emerge relates to PTMs, and while the presence of glycoproteins and phosphoproteins in the CW is now well established (Chivasa et al., 2005; Jamet et al., 2006; Kaida et al., 2010; Melo-Braga et al., 2012; Ruiz-May et al., 2012a) this represents a relatively unexplored area of plant wall biology. Almost nothing is known about the functional significance of such decorations in CW proteins, but suppressing the expression of key enzymes associated with modification of the *N*-glycans (α-mannosidase and β-*N*-acetyl hexosaminidase) in either tomato or pepper (*Capsicum annuum*) has been reported to have profound effects on the ripening (Meli et al., 2010; Ghosh et al., 2011). This therefore represents a potentially exciting area of future research, which will be aided by new analytical pipelines for identifying PTMs and structurally characterizing complex *N*-glycan modifications (Ruiz-May et al., 2012b). An example of such an approach, and the first reported study of the tomato fruit *N*-glycoproteome, was described by Catalá et al. (2011), who used the *N*-glycan binding lectin Concanavalin A, coupled with two-dimensional liquid chromatography, to identify 133 CW proteins from RR stage tomato fruit pericarp. Of these, 89% had a predicted N-terminal secretory SP, suggesting that such as lectin affinity approach both allows a substantial enrichment in CW proteins and provides an opportunity to characterize the sites of *N*-glycosylation and structures of the associated *N*-glycans.

## A COMPARISON OF TOMATO FRUIT CW PROTEIN STUDIES

**FIGURE 1:**
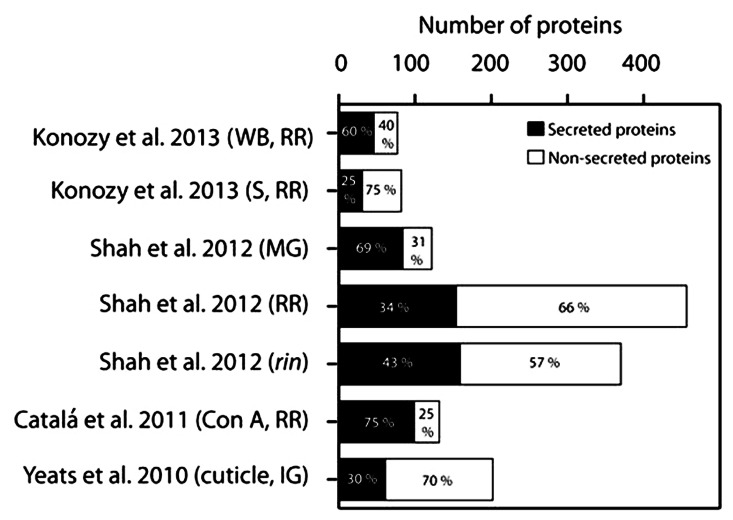
**Analysis of the proportions of proteins identified as entering the secretory pathway, as reported in the cited publications, from immature green (IG), mature green (MG), and red ripe (RR) stage wild type fruit and those of the *rin* mutant, using SignalP 4.1 Server (www.cbs.dtu.dk/services/SignalP). S, soluble; WB, wall-bound**.

**FIGURE 2:**
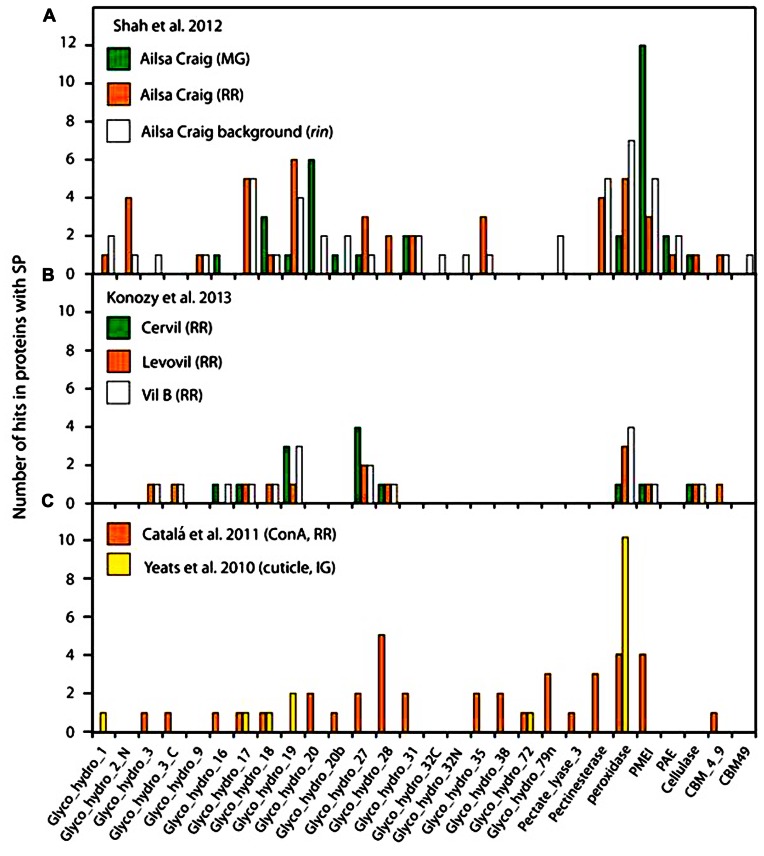
**Classification of tomato fruit proteins with a SP described in the cited publications, generated using the Pfam database (http://pfam.sanger.ac.uk)**. **(A)** Classification of proteins identified in MG stage, RR stage, and *rin* mutant and of tomato fruit infected with *B. cinerea*. **(B)** Classification of proteins identified in RR stage in different cultivars of tomato. **(C)** Classification of proteins in enriched proteome studies targeting the cuticle [immature green (IG) stage] or after using lectin affinity chromatography (RR stage).

Robust bioinformatic tools, such as SignalP 4.1 (Petersen et al., 2011), have been developed to predict the presence of an N-terminal SP and such computational approaches provide a reasonably reliable, although certainly not perfect, indicator of targeting to the secretory pathway. A portion of these secretory proteins eventually traffic to the apoplast, while other subsets localize within various compartment of the endomembrane system, or even other organelles (Rose and Lee, 2010). While a few CW proteins may travel through a non-canonical secretion pathway proteins (Cheng et al., 2009; Rose and Lee, 2010; Zhang et al., 2011), there is little evidence to suggest that these are anything other than rare exceptions. Thus, SP prediction represents a useful “first pass” means to predict the secretome and to estimate the degree of contamination of CW protein extracts with intracellular proteins. We examined the data derived from several of the tomato fruit proteome studies described above using SignalP 4.1, and determined that a relatively high proportion of predicted non-secreted proteins was identified in most cases, including well-known cytosolic proteins, which underscores the extent of the problem of contamination (**Figure 1**). The lectin affinity chromatography (Catalá et al., 2011) yielded the highest percentage of predicted secreted proteins (75%) and thus, by inference, the lowest degree of contamination (**Figure 1**). We note that our analysis used a newer version of the software than was used in the original studies and so the values differ slightly from those that were originally reported. The lowest percentages of predicted CW proteins were evident in studies of the RR stage fruit, where the deterioration in tomato fruit integrity or pathogen mediated tissue damage likely resulted in cell rupture and consequent contamination. Analysis of the functional categories of the tomato fruit CW proteins identified in the various studies suggests that most are associated with CW modifications and they can be assigned to a range of GH families (**Figure 2**). The pectin esterase, pectin methylesterase inhibitor (PMEI) and peroxidase families are particularly well represented, as are members of the GH17 and GH19 families. These correspond to endo-1,3-β-glucanases and chitinases, respectively, which are well-known families of defense-related proteins and accordingly they were particularly prevalent in RR stage tomato fruit challenged with *B. cinerea* (**Figure 2A**; Shah et al., 2012). These defenses-related proteins did not show the same level of representation in tomato cultivars without infection (**Figure 2B**), suggesting that fungal infection triggered the expression of defense-related proteins. Interesting, during the infection with *B. cinerea*, β-galactosidase (GH35 family) was absent in the MG stage but was well represented in RR fruit (**Figure 2A**; Shah et al., 2012). This is in agreement with previous studies where β-galactosidase expression has been observed in the later stages of tomato fruit ripening (Smith et al., 2002; Sozzi et al., 1998). Furthermore, high β-galactosidase activity has been associated with fruit ripening, during which a substantial amount of galactose is lost from the wall (Seymour et al., 1990; Sozzi et al., 1998). In contrast, PMEI was one of the most abundant functional categories in the MG stage but was present at very low levels in both infected (**Figure 2A**) and non-infected (**Figure 2B**) RR stage tomatoes from different cultivars. Moreover, the representation of the PMEI was low in RR fruit even after lectin enrichment of these putative glycoproteins (**Figure 2C**). PMEI proteins were more frequently identified in fruits of the non-ripening *rin* mutant compared with wild type (**Figure 2A**), which further supports the notion that expression declines during ripening. PMEI regulates the expression of pectin methylesterase (PME) enzymes, which catalyze the de-esterification of pectins in the CW during ripening (Prasanna et al., 2007; Reca et al., 2012). This can make pectin polymers more susceptible to hydrolytic depolymerization by PG (Brummell and Harpster, 2001; Wakabayashi et al., 2003; Prasanna et al., 2007). Our analysis suggests that PMEI proteins may suppress the activity of PMEs in MG fruit during *B. cinerea* infection, thereby limiting pectin depolymerization, which in turn may strengthen the CW and deter microbial invasion. Indeed, the infection of MG tomato fruit by *B. cinerea* was limited even though the proportions of defense-related proteins were lower than in RR fruit (Shah et al., 2012).

It is noteworthy that when looking at the various studies as a whole, many known fruit CW proteins were not detected, and those that were identified were generally abundantly expressed. This suggests that additional enrichment and fractionation steps, together with higher sensitivity MS platforms will be necessary to provide more holistic coverage of the fruit CW proteome.

## SUMMARY

Of the various plant subcellular proteomes that have been studied, arguably the most technically challenging is that of the CW, for the reasons described above. Moreover, the CW proteomes of fleshy fruit, such as tomato, represent extreme examples of such challenges, due largely to the composition and properties of the extracellular matrix that often limits effective and representative protein extraction. This raises the question of whether a comprehensive and quantitatively significant assessment of the whole fruit CW proteome is achievable. Remarkably, there are still no reports of large scale proteomic profiling initiatives of the tomato fruit CW spanning the various stage of fruit development and ripening in a single study and this likely reflects the major technical hurdles. The absence of equivalent data sets therefore currently limits biologically informative comparisons between studies (e.g., **Figure 2**). However, “proteomics” comes in many shapes and flavors and recent reports have shown that a great deal of useful information can be learnt from well-established and emerging analytical approaches (Nikolovski et al., 2012; Parsons et al., 2012; Zielinska et al., 2012). A promising emerging area is in the field of PTMs and, in particular, studies of the CW glycoproteome and phosphoproteome will doubtless shed new light on many aspects of fruit biology. This is further suggested by a recent study describing changes in *N*-glycosylation, phosphorylation, and Lys-acetylation during grape berry infection by the pathogen *Lobesia botrana* (Melo-Braga et al., 2012). A similar analysis of such changes during tomato fruit development and responses to environmental changes will doubtless give equivalent information and take analysis of this dynamic subcellular proteome to the next level.

## Conflict of Interest Statement

The authors declare that the research was conducted in the absence of any commercial or financial relationships that could be construed as a potential conflict of interest.

## Abbreviations

CW: cell wall
GH: glycosyl hydrolase
GT: glycosyltransferase
IG: immature green
MG: mature green
PG: polygalacturonase
PMEI: pectin methylesterase inhibitor
PR: pathogenesis related
PTM: post-translational modifications
*rin*: *ripening inhibitor*
RR: red ripe
SP: signal peptide.
